# The Conundrum of the National Exit Test (NExT) in India

**DOI:** 10.7759/cureus.101813

**Published:** 2026-01-18

**Authors:** Nikhil Aggarwal

**Affiliations:** 1 Anatomy, Army College of Medical Sciences, Delhi Cantt, New Delhi, IND

**Keywords:** healthcare, india, medical, national exit test, next

## Abstract

The National Exit Test (NExT) has emerged as a contentious issue in the landscape of Indian healthcare education. With a historical backdrop of evolving medical education and the pressing need for quality healthcare for all, NExT aims to standardize medical graduate competency levels. While the intent is laudable, the proposal has triggered intense debates. NExT's proponents argue that standardization will raise educational quality, ensuring minimum competence among medical professionals. It also seeks to bridge the gap between theory and practice, better preparing graduates for evolving healthcare demands. Additionally, NExT promises equal opportunities for all students, addressing issues of regional disparities and limited seats. However, NExT faces criticism for potentially homogenizing curricula, neglecting specialization, and burdening students and institutions. Drawing lessons from international counterparts, such as the United States Medical Licensing Examination (USMLE) and the United Kingdom Foundation Programme (UKFP), India can strike a balance by incorporating flexibility into assessments and investing in infrastructure. Ongoing evaluation and adaptation of NExT will be crucial to mitigate unintended consequences. As of the most recent update, the NMC has postponed NExT, indicating its significance and complexity. NExT regulations propose a two-phase examination with implications for internships and postgraduate course entrance, aiming to validate graduates' eligibility for medical practice and specialization. In conclusion, India faces the challenge of harmonizing quality and equity in healthcare education through NExT. By learning from global experiences and adopting an inclusive, adaptable approach, India can navigate this conundrum and produce competent healthcare professionals ready to meet the diverse needs of its population.

## Editorial

The National Exit Test (NExT) has emerged as one of the most debated reforms in the history of Indian medical education. Envisioned as a common final examination for all medical graduates, NExT aims to standardize competency levels and ensure minimum acceptable standards of medical practice across the country. While the objective aligns with the broader goal of improving healthcare quality and patient safety, its proposed structure, scope, and implications have generated considerable academic and professional discourse.

India’s medical education system has evolved significantly since its inception during the British colonial era. The post-independence expansion of medical colleges led to a substantial increase in the number of trained doctors, improving access to healthcare education. However, this rapid growth also resulted in marked variability in institutional infrastructure, faculty strength, clinical exposure, and assessment standards. Consequently, concerns regarding uneven graduate competence and quality assurance have persisted, prompting regulatory bodies to explore mechanisms for standardization. The concept of NExT emerged from this backdrop, with the intention of balancing expansion with accountability and uniformity in medical training [1].

Supporters of NExT argue that a national exit examination can serve as an effective quality assurance tool by ensuring that all graduates, irrespective of their institution, meet a defined minimum standard of knowledge and clinical skills. By emphasizing clinically oriented assessment, NExT seeks to bridge the long-recognized gap between theoretical learning and practical application. Furthermore, by acting as a single examination for licensure and postgraduate entrance, NExT is projected to create a more equitable system for career progression, reducing dependence on multiple competitive entrance examinations and addressing disparities arising from regional and institutional differences [2].

At the same time, the proposal has raised substantial concerns among students, faculty, and medical educators. One major criticism relates to excessive standardization and the risk of curricular homogenization. India’s healthcare needs vary widely across regions, and critics argue that a uniform exit examination may inadequately account for local disease patterns, public health priorities, and contextual clinical challenges. There is apprehension that such an approach could limit institutional flexibility, stifle innovation, and undervalue specialized competencies that require focused training beyond general medical proficiency [3].

Another significant concern is the added academic and psychological burden on medical students. Undergraduate medical education in India is already intensive and prolonged, and the introduction of a high-stakes exit examination may exacerbate stress, anxiety, and performance pressure. In parallel, medical institutions may face logistical and infrastructural challenges in implementing NExT, particularly those with limited resources. Without adequate faculty training, assessment preparedness, and infrastructural investment, the transition to a new national examination framework could disrupt existing curricula and training pathways [4].

Insights from international models further highlight the complexity of implementing national exit examinations. The United States Medical Licensing Examination (USMLE) and the United Kingdom Foundation Programme (UKFP) demonstrate how standardized assessments can ensure minimum competence, yet they also reveal limitations. These include an overemphasis on test performance, disproportionate influence on specialty selection and career trajectories, and limited success in addressing workforce distribution, particularly in rural and underserved areas. Such experiences underscore that exit examinations, while valuable, cannot function effectively in isolation from broader educational and health system reforms [5].

In the Indian context, recent regulatory developments reflect recognition of these challenges. On the advice of the Ministry of Health and Family Welfare, the National Medical Commission (NMC) issued a public notice dated 13.07.2023 stating that the implementation of the NExT has been postponed until further orders. Earlier, the NMC had notified the NExT Regulations 2023, proposing a two-phase examination conducted twice a year. As per these regulations, NExT Step 1 would be followed by a compulsory one-year internship, with the Step 1 score being used for postgraduate course ranking. NExT Step 2, to be attempted after completion of internship, would function as the licentiate examination required for registration and practice of contemporary medicine in India. Foreign medical graduates intending to practice in India would also be required to clear NExT Step 1, complete an internship, and pass NExT Step 2 [5].

The NMC has further stated that NExT would serve multiple purposes, including validation of eligibility for medical registration, licensure to practice contemporary medicine, and determination of eligibility and ranking for admission to postgraduate medical education across specialties. This multi-functional role positions NExT as a central regulatory instrument with far-reaching implications for undergraduate training, internship, licensure, and postgraduate education.

In conclusion, the NExT represents a complex and consequential reform in Indian medical education. While its intent to enhance quality and equity is widely acknowledged, its successful implementation will depend on careful planning, phased execution, infrastructural preparedness, and sustained stakeholder engagement. Drawing lessons from international experiences and adapting them to India’s unique healthcare landscape will be essential. A flexible, inclusive, and continuously evaluated approach may help reconcile the dual goals of standardization and diversity, ultimately ensuring the production of competent medical professionals capable of addressing the varied healthcare needs of India’s population.
